# Early clinical efficacy of 3D-printed artificial vertebral body in spinal reconstruction after total en bloc spondylectomy for spinal tumors

**DOI:** 10.1186/s12891-024-08069-7

**Published:** 2024-11-19

**Authors:** Xiaodong Wang, Shaosong Sun, Yuanyuan Jiang, Bao Ren, Xiong Zhang, Jun Miao, Jingtao Ji, Ye Han

**Affiliations:** 1 Department of Orthopaedics, Affiliated Hospital of Hebei University, No.212, Yuhua Road, Baoding City, Hebei 071000 China; 2 Department of Surgery, Affiliated Hospital of Hebei University, Hebei, China; 3 Tianjin Hospital of Tianjin University, Tianjin, China

**Keywords:** Total en bloc spondylectomy, 3D-printed prosthesis, Spinal tumor

## Abstract

**Background:**

Total en bloc spondylectomy (TES) is a recognized surgical approach for managing spinal tumors. With advancements in three-dimensional (3D) printing technology, the use of 3D-printed prosthetics for vertebral reconstruction post-tumor resection has gained traction. However, research on the clinical implications of these prosthetics remains limited.

**Methods:**

This retrospective study evaluated patients who underwent TES for primary and metastatic thoracolumbar tumors at the Department of Spinal Surgery, Tianjin Hospital, between October 2017 and September 2020. These patients received anterior reconstruction with 3D-printed artificial vertebral bodies.

**Results:**

14 patients completed the surgery, with intraoperative blood loss ranging from 1,400 to 4,200 ml (mean 2,767 ± 790 ml) and operative duration between 240 and 520 min (mean 382 ± 75.9 min). The follow-up period extended from 7 to 43 months, with an average of 19.9 ± 9.5 months. Standardized prefabricated prosthetics were utilized in nine patients, while five received customized prosthetics. Throughout the follow-up, there were no reports of posterior connecting rod, 3D-printed prosthetic, or pedicle screw failures. Notably, one patient presented with significant prosthetic subsidence resulting in screw loosening, and three cases of prosthetic subsidence were observed.

**Conclusion:**

The incorporation of 3D-printed prosthetics in TES procedures yielded favorable clinical outcomes. Further research is warranted to optimize these prosthetics for enhanced postoperative stability and patient-specific applications.

## Background

Total en bloc spondylectomy (TES) is a highly effective surgical technique for the management of benign aggressive, primary malignant, and certain metastatic spinal tumors. Its primary objective is the complete resection of the tumor. Initially reported by Tomita in the 1990s, this method has consistently delivered satisfactory outcomes in the treatment of spinal tumors over subsequent decades [1–4]. With advances in targeted and endocrine therapies, the life expectancy of patients with spinal tumors has significantly increased. Concurrently, TES has evolved through various modifications and is presently regarded as a crucial surgical approach in the management of these tumors [5–8]. Contemporary evidence indicates that TES can effectively mitigate neurological symptoms and enhance the quality of life for patients [9].

TES necessitates the excision of the anterior vertebral body, posterior elements, and associated ligamentous structures, significantly compromising spinal stability. This instability requires substantial reconstructive efforts and fixation. Titanium cages, which are currently the predominant choice for anterior spinal reconstruction, present a high incidence of subsidence due to point contact with the vertebral endplates. Notably, severe subsidence may precipitate implant failure through fracture, an issue that becomes more pronounced following the resection of extensive spinal segments [10–13].

Advancements in three-dimensional (3D) printing technology have led to the increased utilization of 3D-printed prostheses for restoring stability after spinal tumor resections, with evidence suggesting their enhanced effectiveness [14–18]. Despite these developments, the literature remains sparse with regard to comprehensive case series analyses. The present study aims to assess the early-term safety and efficacy of 3D-printed vertebral body prostheses in re-establishing anterior spinal stability post-TES for spinal tumors. We hypothesize that the failure rate of 3D-printed artificial vertebrae is low, and subsidence is minimal.

## Methods

This retrospective study focused on patients who underwent total spondylectomy for primary and solitary metastatic thoracolumbar tumors at the Department of Spine Surgery, Tianjin Hospital, from October 2017 to September 2020. These patients received anterior reconstruction using 3D-printed artificial vertebral bodies. The research protocol adhered to the Declaration of Helsinki and received approval from the Ethics Committee of Tianjin Hospital.

Inclusion Criteria (a) Patients diagnosed with either solitary primary spinal tumors or metastatic tumors, confirmed through preoperative X-ray, CT, MRI, and PET-CT examinations. (b) Tumors classified as Type I to V according to the Tomita classification. (c) Patients evaluated preoperatively for metastatic spinal tumor prognosis using the Tokuhashi scoring system, with a predicted survival time exceeding six months. (d) Patients undergoing spinal surgery for the first time.

Exclusion Criteria (a) Patients with incomplete medical records. (b) Patients with a predicted survival time of less than six months. (c) Patients undergoing subsequent spinal surgeries.

This study encompassed a cohort of 14 patients, comprising 10 males and 4 females, whose ages ranged from 15 to 73 years. The sample included 4 cases of primary bone tumors—specifically, 2 cases of giant cell tumors of bone, 1 case of aggressive hemangioma, and 1 case of osteosarcoma. Additionally, there were 10 cases of metastatic tumors: 2 of breast cancer, 2 of renal cancer, 1 of rectal cancer, 2 of thyroid cancer, 2 of lung cancer, and 1 of gastric cancer. Surgical interventions varied, with 10 patients undergoing single-segment total spondylectomy, 2 patients receiving double-segment spondylectomy, and the remaining 2 patients subjected to triple-segment spondylectomy. Detailed demographic and clinical characteristics of the patient cohort are summarized in Table 1.

**Table 1:** The general information of 14 patients underwent TES

No.	Gender	Age	Tumor statue	Tumor location	Tomita Score	Tokuhashi Score	WBB sectors	neoadjuvant therapy	adjuvant therapy
1	F	66	Metastasis	T9	2	9	5–9 C	yes	no
2	M	15	Primary	L4			4–11 D	no	no
3	M	38	Metastasis	T2-T3	5	9	4–10 C	yes	yes
4	F	63	Primary	T6			4–9 C	no	no
5	M	66	Metastasis	T5	2	13	1–12 D	no	no
6	F	43	Metastasis	T10	5	9	6–9 D	yes	yes
7	M	73	Metastasis	T9	5	9	4–9 D	no	yes
8	M	66	Metastasis	T3-T4	5	9	4–12 C	no	no
9	M	30	Primary	T10			5–8 C	no	no
10	F	57	Primary	T10			3–7 C	no	yes
11	M	56	Metastasis	T4-T6	3	10	4–11 C	no	no
12	M	50	Metastasis	L2	3	13	5–8 C	no	yes
13	M	72	Metastasis	T2	2	14	4–9 D	no	no
14	M	63	Metastasis	T4-T6	5	10	3–10 D	no	yes

**Fig. 1:**
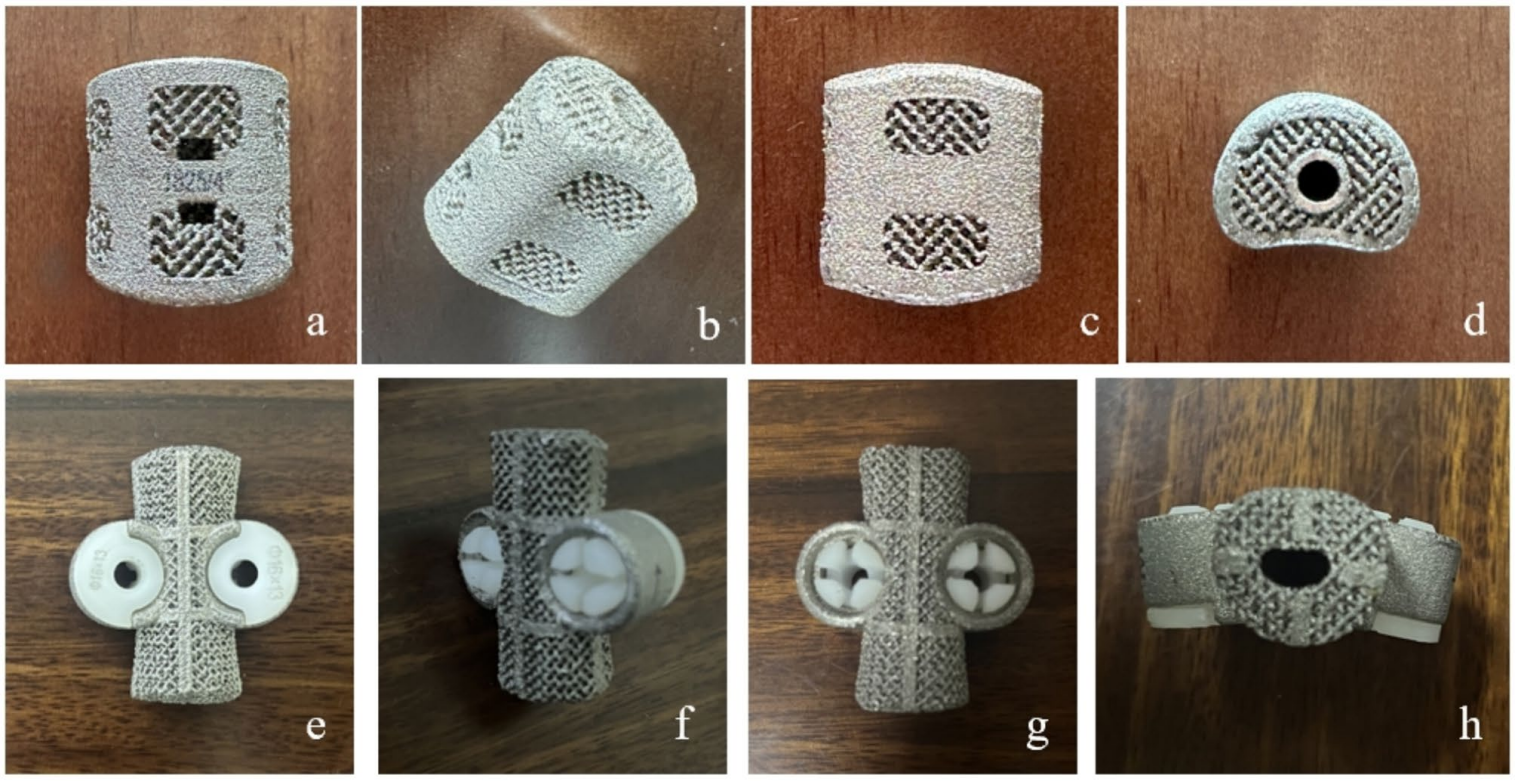
**a**-**d** Prefabricated 3D -printed artificial vertebrae. Front view(**a**), rear view(**b**), side view(**c**), top view(**d**).e-h Customized 3D-printed artificial vertebral bodies (with pedicle screws buckle already installed). Front view(**e**), rear view(**f**), side view(**g**), top view(**h**)

The artificial vertebral bodies used in this group of cases were produced by AK Medical Company (Beijing, China) using 3D printing technology. The artificial vertebral bodies are made of titanium alloy and processed through electron beam melting technology, with a porosity of 80% and pore size of 800 ± 200 μm. The main shape of the artificial vertebral body is cylindrical, available in both prefabricated and customized types. For the prefabricated type, the contact dimensions at both endplates of the thoracic vertebrae are 15 × 21 mm and 18 × 24 mm, for the thoracolumbar segment 30 × 36 mm, and for the lumbar segment 30 × 36 mm. The endplates of the artificial vertebral body are designed with a curved shape, with three design schemes for the angle between the plane and the horizontal plane: 0°, 4°, and 8°. The height ranges from 25 mm to 120 mm, with an interval of 2.5 mm between adjacent models. During surgery, to enhance the stability of the spine, a relatively short artificial vertebral body is generally chosen, but the shortening should not exceed 1/3 of the height of the removed vertebra. Custom artificial vertebral bodies require contacting the relevant engineers to make a bespoke order, with a customization period of 2 weeks. Based on preoperative CT scan results, computer software assists in measuring the height, curvature, and endplate contact angle of the artificial vertebral body. Taking the measured height and adding or subtracting 2 mm, three artificial vertebral bodies are manufactured as backups. Artificial vertebrae can be designed with pre-reserved pedicle or self-stabilizing screw holes as required to enhance the stability of the prosthetic vertebrae (Fig. 1).

### Surgical method

#### Posterior approach surgery

Under general anesthesia, the patient is positioned prone on the operating table. A midline incision is created along the thoracodorsal skin and subcutaneous tissue to expose the bilateral laminae, transverse processes, and approximately 3 cm of rib on each side in the thoracic region, as well as the bilateral laminae and facet joints in the lumbar region. Pedicle screws are strategically placed above and below the affected vertebrae as required.

In the thoracic region, a 3 cm section of rib and the corresponding rib heads are resected and coated with bone wax to prevent bleeding from the rib ends, with meticulous care to avoid injury to the pleura, intercostal nerves, and vessels. The pleura is gently separated from the vertebral bodies bilaterally, with maximal anterior displacement, and gauze is placed anterior to the vertebral body for added protection when necessary.

The inferior half of the lamina of the adjacent normal segment cephalad to the target vertebra is excised. A wire saw is then used to transect the pedicles of the target vertebra bilaterally from beneath the lamina to the intervertebral foramen, thus removing the posterior elements of the diseased vertebra.

In the thoracic segment, ligation of the intercostal nerves and vessels may be performed, while in the lumbar segment, the segmental blood vessels are coagulated to achieve complete isolation of the diseased vertebra from surrounding tissues and anterior abdominal structures.

An “S” shaped retractor is positioned anterior to the vertebral body for protection of the vessels, and a temporary fixation rod is placed unilaterally. The intervertebral discs are addressed by incising the posterior annulus fibrosus bilaterally adjacent to the spinal cord and using a bone chisel to detach the superior and inferior intervertebral discs of the diseased vertebra from posterior to anterior.

Subsequent to meticulous dissection and division of the posterior annulus fibrosus and posterior longitudinal ligament bilaterally towards the midline, the vertebral body is fully detached and rotated out laterally. Remaining disc material from the adjacent vertebral bodies at the cranial and caudal ends of the diseased vertebra is removed, along with the cartilaginous endplates.

A preoperatively designed 3D-printed artificial vertebra, produced by AK Medical Company, is then inserted. Fixation rods are alternately installed bilaterally, followed by mild compression and tightening of the set screws. Intraoperative fluoroscopy with a C-arm X-ray machine is utilized to confirm proper placement of the prosthesis, ensuring appropriate height and size, central alignment, and congruence with adjacent endplates.

After securing hemostasis and placing a drain, the skin incision is meticulously sutured closed.

### Anterior-posterior combined approach in lumbar surgery

For lumbar segments at or below L3, where vertebrae are substantial and challenging to excise solely via the posterior route, we adopted a combined anterior-posterior approach. This facilitated complete spondylectomy and subsequent reconstruction for spinal stability.

Initially, the surgery employed the posterior approach, entailing thorough removal of the posterior elements—akin to pedicle transection and total laminectomy typically performed in posterior procedures. Care was taken to maximally decompress the spinal cord. In cases of adhesion, meticulous dissection was required to detach the vertebrae and neural elements from the posterior side. This phase included severing the posterior annulus fibrosus and the posterior longitudinal ligament of the targeted vertebra, followed by the excision of as much intervertebral disc material as feasible. Posterior pedicle screw-rod fixation was then applied.

Upon completion of the posterior phase, a drainage tube was inserted and the incision sutured. The patient was repositioned supine for the anterior phase. An extraperitoneal approach via a midline abdominal incision was executed, retracting the anterior vasculature laterally to reveal the tumorous vertebra. The anterior longitudinal ligament, annulus fibrosus, and adjacent intervertebral disc material were resected, allowing for complete anterior removal of the affected vertebra. The cartilaginous endplates near the targeted segment were addressed to expose the bony endplates, whereupon a custom 3D-printed vertebral prosthesis was implanted. Specialized anterior prosthesis screws were then placed.

Following meticulous inspection to confirm no damage to adjacent vessels or organs, a drainage tube was placed, and the incision was meticulously closed in layers.

### Postoperative management

Following surgery, all patients were mandated to adhere to bed rest and commenced ambulatory activities after one week, with the assistance of a rigid thoracolumbar brace, under physician supervision. The surgical drainage apparatus was removed once the daily volume of postoperative drainage fell below 50 mL. For three months subsequent to the operation, patients were instructed to wear the rigid brace, which was then replaced with a soft lumbar support to be used until the completion of six months postoperatively.

In the postoperative care regimen, patients were prescribed the nonsteroidal anti-inflammatory drug Celecoxib, at a dosage of 200 mg twice daily, for a duration of three weeks. Additionally, prophylactic anticoagulation with low molecular weight heparin was administered subcutaneously at a dose of 5100 IU once daily for a period of two weeks to mitigate the risk of thromboembolic events.

### Follow-up content

The surgical duration, intraoperative blood loss, postoperative complications, and Visual Analogue Scale (VAS) scores were meticulously recorded preoperatively, as well as at 3 and 6 months postoperatively. Neurological function was assessed using the Frankel grading system preoperatively, as well as at three and six months following surgery. Preoperative evaluation included X-ray and computed tomography (CT) imaging for all patients, with these assessments scheduled to be repeated at three and six months after surgery, and subsequently every six months. The CT scans were utilized to evaluate the positioning, subsidence, and to detect any potential loosening or migration of the 3D-printed prostheses.

### Imaging and clinical assessment

All preoperative patients were subject to diagnostic imaging, including X-ray, computed tomography (CT), magnetic resonance imaging (MRI), and whole-body bone scans. In instances of metastatic spinal tumors, Tomita scores varied from 2 to 5, with a mean score of 3.7. Tokuhashi scores ranged between 9 and 14, averaging 10.5. Fourteen patients exhibited symptoms of spinal cord or nerve root compression to varying extents, with three cases resulting in paraplegia and eleven cases manifesting as intercostal neuralgia or diminished strength in the lower limbs. All patients reported experiencing some degree of pain in the thoracic, thoracolumbar, or lumbar regions.

The intensity of preoperative and postoperative pain was quantified using the Visual Analogue Scale (VAS), while neurological function was assessed via the Frankel grading system. Prosthetic subsidence, identified through X-ray or CT, was defined as a reduction of at least 2 mm. In cases involving vertebral resection and stabilization reconstruction, the restoration of sagittal alignment was indicated by changes in the Cobb angle. This angle is delineated by the upper endplate of the vertebra immediately superior and the lower endplate of the vertebra immediately inferior to the affected region, measured both before and after surgery. The percentage of kyphosis correction was calculated using the formula: Kyphosis correction rate (%) = [(Preoperative local kyphotic angle - Postoperative kyphotic angle) / Preoperative local kyphotic angle] × 100%.

### Statistical methods

Statistical analyses were conducted utilizing SPSS version 26.0 (IBM, Armonk, NY, USA). The results are reported as means ± standard deviations. Comparison of the VAS scores was achieved through a paired t-test. A p-value below 0.05 was deemed to indicate statistical significance.

## Results

### General conditions

All 14 patients successfully underwent surgical procedures. Thirteen cases were treated using a posterior total en bloc spondylectomy (TES), while one case required a combined anterior-posterior approach. Intraoperative blood loss varied from 1,400 to 4,200 ml, with a mean of 2,767 ± 790 ml. Surgical duration ranged from 240 to 520 min, averaging 382 ± 75.9 min. The postoperative hospital stay spanned 9 to 17 days, with a mean duration of 12 ± 2.4 days. Follow-up for all patients extended from 7 to 43 months, with an average follow-up of 19.9 ± 9.5 months(Table 2).

**Table 2:** Treatment and outcomes of 14 patients who underwent TES

No	Operative Time(min)	Blood loss (ml)	Approach	Pathology	Tumor status	Follow Up(month)
1	360	2400	Posterior	Breast cancer	AWD	16
2	382	2600	Posterior- Anterior	Giant cell tumor of bone	NED	23
3	430	4000	Posterior	Lung cancer	AWD	28
4	260	2250	Posterior	Aggressive hemangioma	NED	43
5	390	2200	Posterior	Thyroid cancer	DOD	22
6	370	2000	Posterior	Breast cancer	DOD	23
7	345	2700	Posterior	Gastric cancer	DOD	30
8	415	3500	Posterior	Lung cancer	AWD	18
9	240	1400	Posterior	Giant cell tumor of bone	NED	20
10	408	2500	Posterior	Osteosarcoma	DOD	16
11	520	4200	Posterior	Kidney cancer	AWD	13
12	358	2600	Posterior	Rectal cancer	NED	12
13	370	2800	Posterior	Thyroid cancer	NED	8
14	500	3600	Posterior	Rectal cancer	NED	7

NED, no evidence of disease; AWD, alive with disease; DOD, dead of disease

In terms of prosthetic use, nine cases involved standardized prefabricated prostheses, while five cases utilized custom prostheses. The latter included one with anterior self-stabilizing screws and four with artificial pedicle screw prostheses. Posterior fixation with pedicle screws was employed in all cases.

For the ten cases involving a single segmental resection, eight used two pairs of pedicle screws for fixation above and below the resected segment. Of these, one incorporated a custom prosthesis with an artificial pedicle screw structure. One case, due to significant osteoporosis, required three pairs of pedicle screws for additional support. Another case involved a custom prosthesis fixed anteriorly with four screws through the prosthesis and posteriorly with a pair of pedicle screws above and below the resected segment.

In cases where two vertebral bodies were excised, custom prostheses with designated pedicle screw positions were used. These were connected to posterior rods and secured with two pairs of screws flanking the resected segments. For patients with three vertebral segments removed, one case utilized a custom prosthesis with two pairs of artificial pedicle screw structures and posterior fixation to two segments above and below the resected segments. Another case employed a prefabricated prosthesis, with fixation achieved using pedicle screws across three segments above and below the resected segment.

Clinical outcomes upon discharge indicated that all patients experienced alleviation of radicular neuropathic pain following comprehensive spinal decompression. At discharge, complete remission of radicular pain was observed in five patients, while nine others still presented with varying extents of neurological symptoms. Three months post-surgery, the Frankel classification for neurological function was as follows: Grade E in eight patients, Grade D in two, Grade C in two, Grade B in one, and Grade A in one patient. Of the 11 patients presenting with reduced muscular strength at baseline, 10 exhibited an improvement of at least one Frankel grade at the three-month follow-up. Notably, one patient advanced from Grade A at admission to Grade D postoperatively. The comparison between preoperative and three-month postoperative neurological status revealed a statistically significant enhancement.

At the six-month follow-up, nerve symptoms had fully resolved in five patients, while another five exhibited recovery with some persistent muscle weakness or pain, representing a significant improvement from their preoperative condition. One patient with complete paraplegia experienced no alleviation of nerve symptoms.

The mean preoperative Visual Analog Scale (VAS) score was 6.4 (SD ± 2.8). At three and six months post-surgery, the mean VAS scores decreased to 1.8 (SD ± 1.5) and 1.1 (SD ± 1.2), respectively, demonstrating statistically significant improvement (*p* < 0.05). Table 3 presents a summary of the VAS scores and Frankel classifications for the cohort.

**Table 3:** Frankel classifications and VAS score

Frankel Score	Preoperation	3 month	6 month
A	3	1	1
B	1	1	0
C	3	2	2
D	4	2	3
E	4	8	8
VAS Scores	6.4(± 2.8)	1.8(± 1.5)	1.1(± 1.2)

At the last follow-up, four patients had succumbed to multi-organ failure caused by tumor metastasis. Throughout the observation period, four additional patients developed new visceral or bone metastases yet survived with their tumors, whereas three patients exhibited no signs of metastatic lesions and lived without tumor recurrence. 

Three cases were diagnosed as aggressive benign tumors, these patients also remained tumor-free. There was no evidence of local recurrence at the surgical sites among any of the patients.

### Imaging results

Imaging follow-up disclosed that the mean preoperative kyphotic Cobb angle of the thoracic spine was 15.0 ± 6.5°, which improved to 9.8 ± 4.5° post-surgery, reflecting a correction rate of 33.3 ± 22%. Two patients with lumbar spine conditions preserved their original lordotic angles, achieving favorable outcomes (Table 4).

**Table 4:** Radiologic outcomes of 14 patients who underwent TES

No	Fixation level	Height of AVB (mm)	Subsidence (mm)	Pre-operative Cobb angle (°)	Postoperative Cobb angle (°)
1	T6-T12	30	-	15.4	14.8
2	L3-L5	52	2	8.8	6.7
3	C7-T5	50	-	22.5	7.0
4	T4-T8	30	-	16.2	15.8
5	T3-T7	30	2	12.6	6.8
6	T8-T12	33	-	3.9	3.2
7	T7-T11	33	5	10.8	4.6
8	T1-T6	50	-	11.7	4.6
9	T8-T12	35	-	17.4	13.9
10	T8-T12	33	-	15.4	14.9
11	T1-T9	70	-	19.3	13.3
12	T12-L4	45	-	10.5	6.4
13	C7-T4	30	-	30.6	17.3
14	T2-9	70	-	15.4	8.0

AVB artificial vertebral body

During follow-up, there were no instances of posterior connecting rod breakage, fractures of 3D-printed implants, or dislodgements. Additionally, there were no reports of pedicle screw breakage or pullout. However, one patient did experience screw loosening attributed to significant implant subsidence. Three patients exhibited implant subsidence, all of which occurred at the upper endplate of the vertebral body beneath the implant.

One case involved implant tilting and a subsidence of approximately 5 mm one month postoperatively, which was linked to intraoperative endplate damage. Despite this, the implant stabilized and showed no further subsidence after six months of follow-up. Imaging conducted 20 months postoperatively confirmed the implant’s stability, though the patient experienced mild thoracic and back pain. This patient passed away 30 months post-surgery due to tumor progression and multi-organ failure.

The remaining two cases showed uniform subsidence of approximately 2 mm, but the implants remained stable over follow-up periods of 22 and 43 months, respectively. These patients reported only minor pain, which did not interfere with their normal activities. All three instances of subsidence involved prefabricated implants; none of the patients experienced screw breakage or required additional surgery.

### Complications

During the surgical procedures, pleural rupture was encountered in six patients, all of whom underwent successful intraoperative repair. Postoperative closed thoracic drainage was not routinely utilized. However, two patients developed postoperative respiratory distress and chest tightness. Subsequent ultrasound examinations revealed pleural effusions, necessitating the placement of closed thoracic drains. These drains were subsequently removed on postoperative days five and seven, respectively.

Intraoperative dural injuries with cerebrospinal fluid leakage occurred in three patients. In two of these cases, the dural tears were sutured, and the muscle and deep fascial layers were securely approximated during wound closure. Additionally, a drainage tube was left in situ for an extended duration to manage the leakage. This tube was removed once the output decreased to less than 50 ml.

One patient experienced delayed wound healing, which resolved by the 20th postoperative day with conservative management, including regular dressing changes. Postoperatively, there were no reports of exacerbated neurological damage or significant vascular injuries among the patients.

### Typical case

#### Case 1

**Fig. 2:**
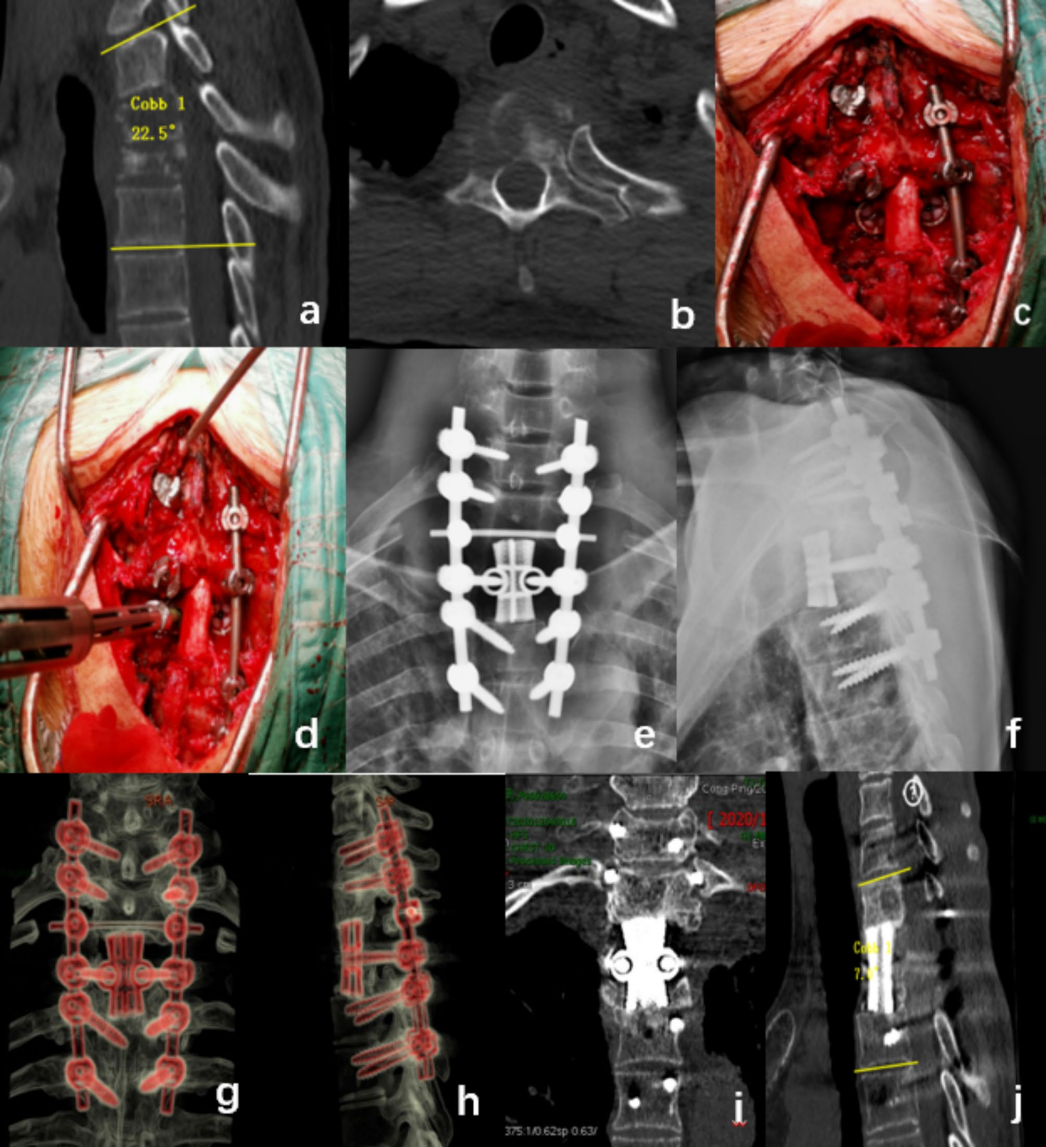
a 38-year-old male patient with pulmonary cancer exhibiting T2 and T3 metastases. Preoperative CT scans of the patient revealed a kyphotic deformity of the thoracic spine (**a**.**b**). During surgery, a customized artificial vertebral bodies is used to replace the excised vertebrae (**c**.**d**). The postoperative X-ray shows that the artificial vertebral body is well positioned (**e**.**f**). 28 months post-surgery, the X-ray and CT images show that the artificial vertebral body is well-positioned without any subsidence or loosening

A 38-year-old male with lung cancer presented with thoracic and dorsal back pain, accompanied by kyphotic deformity, and was diagnosed with T2 and T2 spinal metastases. The patient underwent a posterior TES surgery with the use of a 3D-printed prosthesis, during which the tumor was completely excised. Postoperative results showed a reduction of 15.5° in the Cobb angle and significant alleviation of pain. At a 28-month follow-up, no local recurrence was observed. The 3D-printed prosthesis and internal fixation remained in good position (Fig. 2).

#### Case 2

**Fig. 3:**
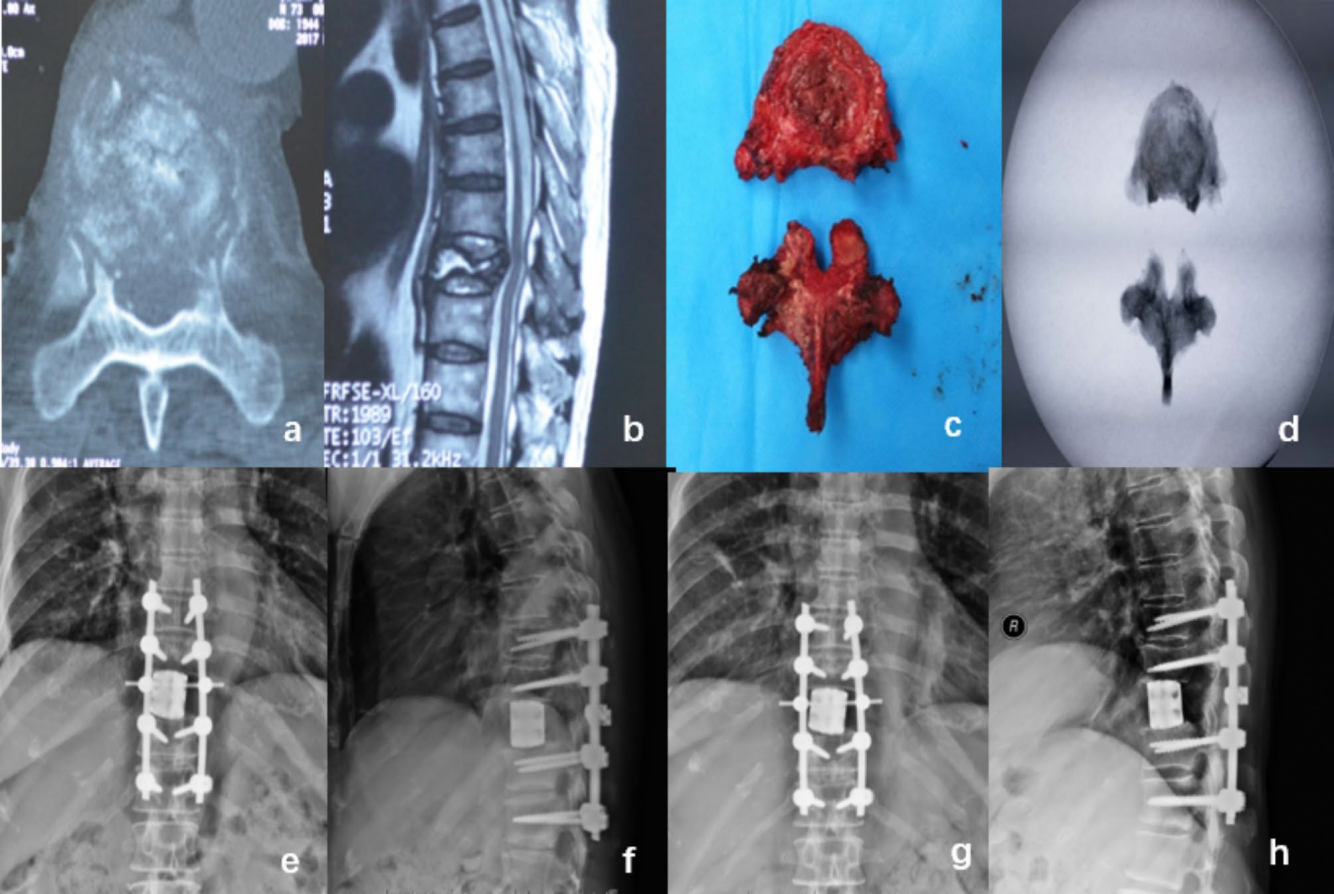
a 66-year-old female patient presented with breast cancer that had metastasized to the T9 vertebra. Preoperative CT and MRI scans disclosed a local posterior convex deformity and osteolytic destruction (**a**.**b**). The affected vertebra was completely excised during surgery(**c**.**d**). postoperative X-rays demonstrated the successful implantation of a prefabricated 3D-printed prosthesis, with notable restoration observed in the sagittal sequence (**e**.**f**). Follow-up X-rays conducted 16 months postoperatively showed no subsidence of the prosthesis(**g**.**h**)

A 66-year-old female with breast cancer presented with metastasis to the T9 vertebra. Preoperative examination revealed osteolytic destruction of the T9 vertebra. During surgery, a prefabricated 3D-printed prosthesis was used to replace the excised vertebra. The patient’s preoperative Frankel Score was C, and it improved to E three months postoperatively. The preoperative Visual Analogue Scale (VAS) score was 6, which decreased to 2 three months after the surgery. During a postoperative follow-up at 16 months, the 3D-printed vertebral prosthesis was well-positioned with no evidence of subsidence (Fig. 3).

#### Case 3

**Fig. 4:**
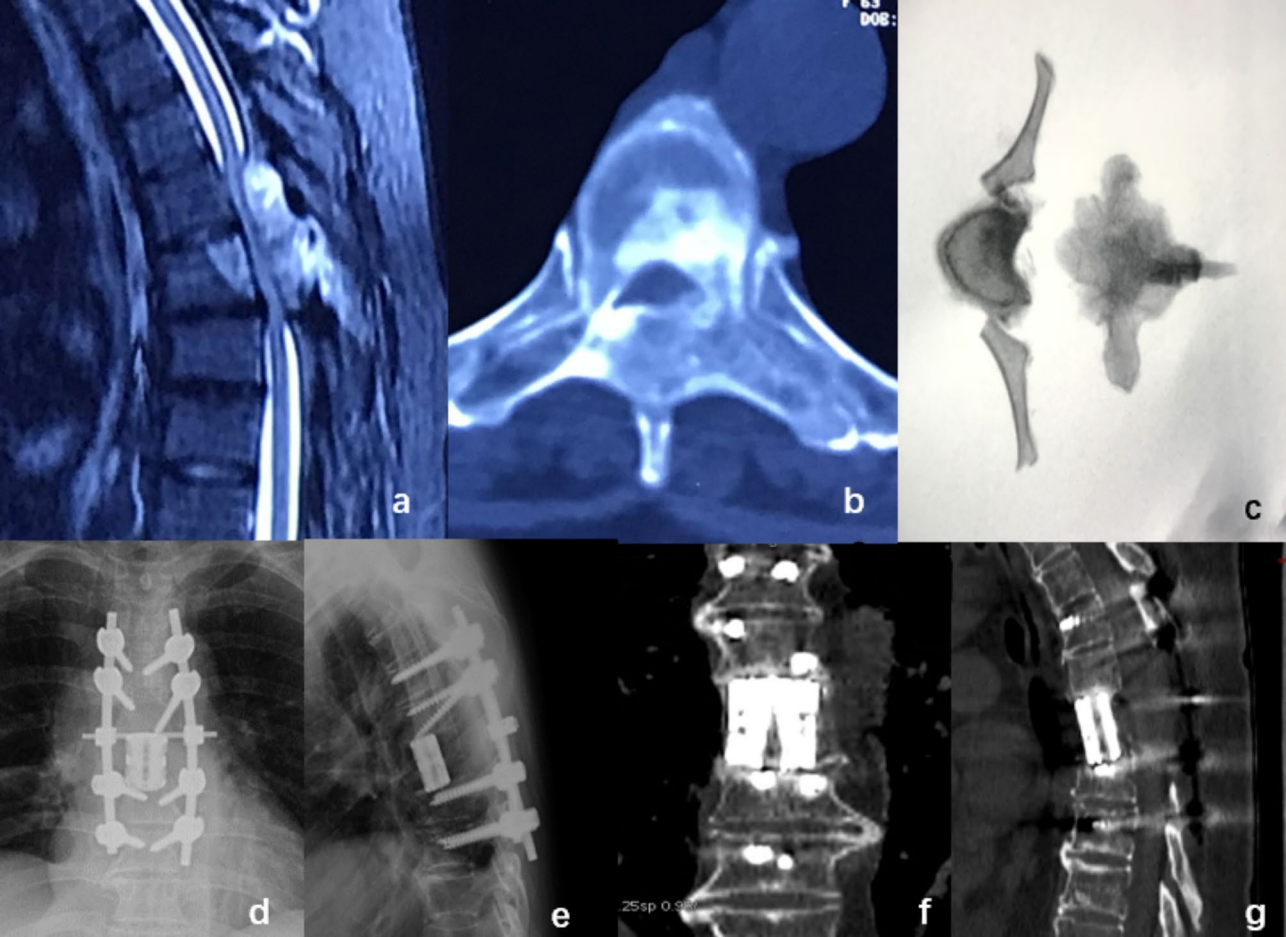
A 63-year-old female patient presented with a thoracic 6 invasive hemangioma. Preoperative CT and MRI scans revealed significant spinal cord compression at the affected segment (**a**.**b**). During surgery, the tumor vertebra was completely excised(**c**). Postoperative X-ray images showed the prefabricated 3D-printed prosthesis was well-positioned (**d**.**e**). CT scans at 43 months postoperatively indicated good adherence of the prosthesis to the adjacent endplates, with a subsidence of 2 mm, no screw loosening, and no internal fixation breakage(**f**.**g**)

A 63-year-old female presented with an aggressive hemangioma at T6, experiencing localized pain. Preoperatively, the patient had a Frankel Score of B and a VAS score of 7. Imaging studies revealed compression of the local spinal cord. A prefabricated 3D-printed implant was used to perform a TES during surgery. Three months postoperatively, the patient’s Frankel Score improved to D, and the VAS score decreased to 2. X-rays showed a 2 mm subsidence of the implant. At a 43-month follow-up, there was no further subsidence of the implant, and the position of the internal fixation was satisfactory. The patient’s pain symptoms had completely resolved (Fig. 4).

## Discussion

The advent of 3D printing technology, which has garnered considerable interest in recent years, involves the transformation of titanium alloy powder into cylindrical implants with specific porosity and pore dimensions using electron beam melting technology. The incorporation of 3D-printed vertebral bodies in spinal surgery is on the rise.

Xu et al. documented the inaugural instance of a bespoke prosthesis in a 12-year-old patient with Ewing’s sarcoma at the C2 level, noting no recurrence or prosthesis subsidence at the one-year mark [19]. Wei et al. described successful long-segment L1-3 giant cell tumor excision and reconstruction using a 3D-printed prosthesis, with maintained stability and no subsidence at an 8-month review [17]. Choy et al. recounted a primary tumor resection at T9 with subsequent 3D prosthesis reconstruction, observing the prosthesis well-placed and signs of osseointegration at 6 months post-surgery [15]. A literature review by Girolami et al. encompassing 13 cases of thoracolumbar tumor resection and 3D prosthesis reconstruction revealed the prostheses’ proficient sagittal alignment capabilities. Although all prostheses exhibited minor settling into adjacent vertebrae, only one case necessitated revision surgery due to significant subsidence causing fixation failure [14]. Current research supports the efficacy of 3D-printed prostheses in spinal tumor interventions [20].

In our study, a single patient exhibited internal fixation loosening, yet no additional surgery was warranted in the absence of symptomatic discomfort. Although three cases showed prosthesis subsidence at the one-month postoperative evaluation, there was no progression of subsidence in later follow-ups, suggesting that the prostheses retained adequate supportive function.

This study utilized two types of artificial vertebral bodies: custom-made and prefabricated prostheses. We employed five custom-made and nine prefabricated artificial vertebral bodies. Prior research indicates that custom-made prostheses can suffer from fit issues. For instance, Chen et al. described a case where a prosthesis was excessively long due to discrepancies between preoperative planning and intraoperative findings, necessitating the modification of an adjacent vertebra [16]. Similarly, Hu et al. reported on eight 3D-printed prostheses, with one being undersized relative to preoperative estimations, leading to a mismatch with the bone defect, and another being oversized, which prompted the use of an expandable prosthesis instead [21]. Hence, it is advisable to have conventional instruments on hand as a contingency when implanting 3D-printed prostheses. In contrast, our study did not encounter any fit issues with the custom-made prostheses.

It is important to recognize that the production of custom prostheses typically requires 2–3 weeks, potentially impacting the recovery of nerve function in patients with deteriorating neurological symptoms. Nevertheless, custom prostheses offer significant benefits, such as an improved match with vertebral endplates, thereby increasing the contact area with the bone and facilitating fusion. Additionally, custom prostheses can be designed with more anchoring points or designated spaces for pedicle screw insertion, enhancing implant stability. In our research, the custom prostheses included space for pedicle screw insertion, which allowed for integration with the posterior rod system, thus bolstering stability.

Regarding the nine prefabricated prostheses used in our study, seven were designated for single-segment thoracic vertebra resection. These prostheses, based on preoperative radiological assessments, were 30–33 mm in height and featured contoured surfaces to adapt to thoracic vertebral endplates. For full vertebral resections in the less complex thoracic and lumbar regions, we advocate for the selection of prefabricated prostheses based on imaging and physical specimen measurements to minimize preoperative delays. This approach is particularly advantageous for patients with rapidly progressing tumors and severe neurological symptoms.

Some literature suggests that a moderate reduction in vertebral body height may enhance spinal stability [22]. Consequently, in our study, the prefabricated prostheses were marginally shorter than the height of the resected area, which included the vertebra and adjacent intervertebral disc. By employing compression fixation with the posterior rod, we posit that this technique can improve stability and augment the contact area between the prosthesis and the bone.

Postoperative failure of internal fixation is a prevalent complication following TES, which may lead to local discomfort and neurological deficits. Effective reconstruction of the anterior spine can significantly reduce the occurrence of internal fixation failure. There are many alternative devices available, such as bone grafts, expandable artificial vertebral bodies, carbon fiber cages, titanium cages, and so on. Among these, bone grafts are less costly and have fewer rejection reactions, but due to their lower strength, they often require additional anterior plate fixation. The height of expandable titanium cages can be adjusted during placement, but the space for filling bone materials is limited [23]. Carbon fiber cages can reduce artifacts under X-ray and CT examinations and have less impact on postoperative radiotherapy, but there is still a lack of long-term clinical follow-up, and the costs are high [24, 25]. Titanium cages are one of the most commonly used vertebral body replacements. Matsumoto and colleagues identified subsidence exceeding 5 mm in titanium cages as a significant contributor to fixation failure [11]. Park [26] and Sciubba [27] observed high rates of internal fixation fractures and failures, 37.5% and 39.1% respectively, in their research utilizing titanium mesh cages for anterior column support in total spinal resections. They attributed fixation failure primarily to nonunion of anterior support structures and recommended vigilant preservation of endplate integrity to mitigate anterior prosthesis subsidence.

Dong et al. reported that artificial vertebral bodies exhibited reduced rates of prosthesis subsidence compared to titanium cages [28]. Similarly, Sun and colleagues documented no instances of prosthesis subsidence when employing 3D-printed prostheses for multi-segment spinal tumor management [29]. In our own research, we observed no rod fractures, but one case of a 5 mm prosthesis subsidence occurred due to intraoperative endplate injury, resulting in screw loosening. The affected patient, experiencing only mild back pain, did not require reoperation.

Previous studies have indicated that the elastic modulus of titanium cages is greater than that of bone tissue. These cages primarily interface with the bone at their ends and endplates, and insufficient contact may precipitate cage subsidence, impede osseous integration, and ultimately cause fixation failure [30, 31]. In contrast, artificial vertebral bodies, designed with porosity and an expanded prosthesis-bone contact surface, enhance bone cell infiltration. The literature suggests that the overall porosity of titanium metal ranges from 60 to 80%, achieving an elastic modulus that closely approximates that of natural bone and diminishes subsidence through stress shielding. Optimal bone cell ingrowth, which is crucial for bone healing, is facilitated by pore sizes of 200–1000 microns [32, 33].

The 3D-printed prostheses utilized in our study boasted a porosity of 80% and a pore size of 800 microns, theoretically offering robust bone support and ingrowth potential [34, 35]. Although our clinical outcomes were promising, the evaluation of metal prosthesis integration with bone remains a challenge, primarily due to examination techniques and the presence of metal artifacts. Consequently, fusion rates were not assessed in our study. Determining accurate fusion rates necessitates larger cohorts and extended follow-up durations.

This study possesses several limitations. Primarily, it is retrospective in nature, focusing exclusively on the outcomes of using 3D-printed artificial vertebral bodies in TES surgeries at a single center. Additionally, the follow-up duration was brief, necessitating further investigation to ascertain long-term effects. Moreover, the limited sample size could potentially bias the findings. Nevertheless, the preliminary evidence suggests that artificial vertebral bodies demonstrate beneficial outcomes in TES procedures, and the employment of 3D-printed artificial vertebral bodies in spinal surgeries appears to hold considerable promise.

## Conclusion

In TES surgery, the use of 3D-printed prosthetics for anterior reconstruction can achieve spinal stability. The improvement of spinal kyphotic deformity is favorable. Despite these promising outcomes, there is a pressing need for prospective, multicenter studies with extended follow-up periods to more conclusively determine the fusion rates and assess the long-term efficacy of 3D-printed prostheses.

## Data Availability

The datasets used and/or analysed during the current study available from the corresponding author on reasonable request.
